# Genome Sequence of the *Salmonella enterica* subsp. *enterica* Serovar Namur Strain 05-2929, Lacking the *Salmonella* Atypical Fimbrial Operon

**DOI:** 10.1128/genomeA.00299-14

**Published:** 2014-04-10

**Authors:** Elodie Barbau-Piednoir, Sophie Bertrand, Nancy H. Roosens, Sigrid C. J. De Keersmaecker

**Affiliations:** a Platform Biotechnology and Molecular Biology, Scientific Institute of Public Health, Brussels, Belgium; b Scientific Service Food-Borne Pathogens, Scientific Institute of Public Health, Brussels, Belgium; c Laboratory of Food and Environmental Microbiology, Earth and Life Institute, Université catholique de Louvain, Louvain-la-Neuve, Belgium; d National Reference Centre for *Salmonella* and *Shigella*, Scientific Service Bacterial Diseases, Scientific Institute of Public Health, Brussels, Belgium

## Abstract

This paper announces the genome sequence and annotation of *Salmonella enterica* subsp. *enterica* serovar Namur strain 05-2929. *S.* Namur is a new serovar (39:z4,z23:−) that was isolated from a patient with salmonellosis in 2005 in Namur, Belgium, and has been identified as lacking the *Salmonella* atypical fimbrial (*saf*) operon.

## GENOME ANNOUNCEMENT

*Salmonella* is one of the major causes of food-borne zoonoses. Between 90,000 and 135,000 cases per year of human salmonellosis were reported in Europe in the period 2008 to 2012 (1). The genus *Salmonella* includes 2 species, i.e., *S. enterica* and *S. bongori*, which are both pathogenic for humans (2). *S. enterica* is further divided into 6 subspecies and 2,587 serovars, which are often named according to the place of first isolation. The serovars are unequally distributed, since 1,547 belong to the subspecies *S. enterica* subsp. *enterica* (subsp. I) (2), which is most often found in human and food samples (1).

In 2005, a *Salmonella* strain (05-2929) was isolated at the Centre Hospitalier Régional de Namur, Belgium, from a stool sample of a patient with salmonellosis. The serotyping of this strain was performed at the Belgian National Reference Center for *Salmonella* and *Shigella*. It was characterized as a *Salmonella enterica* subsp. *enterica* (subsp. I) serovar, presenting a new antigenic formula, i.e., 39:z4,z23:−. This was confirmed by the World Health Organization (WHO) Collaborating Centre for Reference and Research on *Salmonella*, Pasteur Institute, Paris, France. This new serovar was added to the White-Kauffmann-Le Minor scheme in 2010 as the *S.* Namur serovar (2).

During the selectivity test of the validation of SYBR green real-time PCR (qPCR) assays for detection in food samples of *Salmonella* spp. based on genus-, species-, and subspecies-specific marker genes, genomic DNA isolated from this *S.* Namur serovar was identified as not amplified by two qPCR assays targeting *safC* (3). *safC* belongs to the *Salmonella* atypical fimbrial (*saf*) operon (4) and was reported to be specific for *S. enterica* subsp. *enterica* (5).

To further characterize this *S.* Namur 05-2929 strain, whole-genome sequencing was performed on an Illumina HiSeq 2000 run using a paired-end and a 4-kbp insert mate-pair library (performed by BaseClear B.V., Leiden, the Netherlands). Sequencing yielded 4,901,007 paired-end and 11,099,754 mate-pair reads (50 cycles, 439-fold coverage), which were assembled *de novo* using the *de novo* assembly option of the CLC Genomics Workbench version 5.1 (CLC bio). The assembly generated 21 scaffolds consisting of 95 contigs, with a maximum scaffold size of 3,131,320 bp and a minimum size of 323 bp. The total sequence length is 4,842,244 bp and has a G+C content of 51.96%.

Genome annotation was performed by the NCBI Prokaryotic Genome Annotation Pipeline (2013) and predicted 4,616 genes, including 4,501 protein-coding genes. This annotation confirmed the deletion of the entire *saf* operon in this *S.* Namur strain. Strains of other subspecies I serovars lacking *safC* have been reported previously (6). Moreover, based on the presence and absence of clade-specific genes and previously identified single nucleotide polymorphisms (7), this *S.* Namur strain can be further classified as an *S. enterica* subsp. *enterica* clade A strain.

It would be interesting to further investigate these deletion and diversification events using the newly available *S.* Namur sequence and to extend this analysis to other strains of the *S.* Namur serovar.

### Nucleotide sequence accession numbers.

This whole-genome shotgun project has been deposited at DDBJ/EMBL/GenBank under the accession no. AWGG00000000. The version described in this paper is version AWGG01000000.
